# NCAPG2 promotes tumour proliferation by regulating G2/M phase and associates with poor prognosis in lung adenocarcinoma

**DOI:** 10.1111/jcmm.13010

**Published:** 2016-11-15

**Authors:** Ping Zhan, Guang‐min Xi, Bin Zhang, Ying Wu, Hong‐bing Liu, Ya‐fang Liu, Wu‐jian Xu, Qingqing Zhu, Feng Cai, Ze‐jun Zhou, Ying‐ying Miu, Xiao‐xia Wang, Jia‐jia Jin, Qian Li, Tang‐feng Lv, Yong Song

**Affiliations:** ^1^ Department of Respiratory MedicineJinling HospitalNanjing University School of MedicineNanjingChina; ^2^ Department of Respiratory MedicineNanjing Chest HospitalMedical School of Southeast UniversityNanjingChina; ^3^ Department of GastroenterologyThe Affiliated Drum Tower Hospital of Nanjing University, Medical SchoolNanjingJiangsuChina

**Keywords:** NCAPG2, lung adenocarcinoma, prognosis, proliferation, cell cycle arrest

## Abstract

NCAPG2 is a component of the condensin II complex and contributes to chromosome segregation *via* microtubule–kinetochore attachment during mitosis. It is well known that NCAPG2 plays a critical role in cell mitosis; however, the role of altered NCAPG2 expression and its transcriptional regulatory function in cancer development remains mostly unknown. Here, for the first time we reported that NCAPG2 was evidently increased in non‐small cell lung cancer tissues compared to adjacent normal lung tissues. Clinicopathological data analysis showed that NCAPG2 overexpression was significantly correlated with lymph node metastasis and pathologic‐Tumour Nodes Metastasen stages, and was an independent prognostic factor in lung adenocarcinoma patients. Moreover, siRNA‐mediated knockdown of NCAPG2 could inhibit tumour cell growth of lung adenocarcinoma cells (A549 and H1299) *in vitro* and could significantly lead to cell cycle arrest in the G2 phase. Furthermore, we found that NCAPG2 silencing significantly decreased the expression levels of G2/M phase cell cycle‐related protein expressions (Cyclin B1, Cdc2) and increased the expression levels of p27 and p21 through Western blot analysis. Taken together, we demonstrated that increased NCAPG2 expression could regulate cell proliferation and identified as a poor prognostic biomarker in lung adenocarcinoma.

## Introduction

Lung cancer is the most common cancer and the leading cause of tumour‐related death 1. In China, lung cancer is estimated to account for 21.6% of all cancer deaths in 2015^2^. Non‐small cell lung cancer (NSCLC) accounts for approximately 80–85% of all lung cancers. Non‐small cell lung cancer can be classified into two common subtypes, adenocarcinoma (AD) and squamous cell carcinoma, which account for approximately 40% and 25–30% of all lung cancers respectively 1. Although prognosis of patients can be improved through effective treatment, the 5‐year survival rate of patients with advanced lung cancer is only 10–15% 3. The extremely poor prognosis of NSCLC is largely because of a high rate of distant metastasis after resection. Thus, it is imperative to identify novel biomarkers and a potential therapeutic target, to improve the clinical treatment of NSCLC patients.

Mitosis is a highly regulated process characterized by dramatic and coordinated morphological changes to ensure the fidelity of chromosome segregation 4. Chromosome condensation during mitosis is critical for proper bi‐oriented chromosome separation 4, 5. The condensin II complex regulates chromosome condensation and segregation during mitosis 6. Recently, several studies found that Condensin II complex also regulates a variety of cellular functions, including chromosome segregation, DNA repair, sister chromatid resolution, gene expression regulation and histone modulation 7, 8, 9, 10, 11. NCAPG2, NCAPD3 and NCAPH2 are components of Condensin II complex 5, 12, 13.

In 2014, Kim *et al*. 14 found that NCAPG2 interacts with Polo‐like kinase 1 (PLK1) during the prometaphase–metaphase transition during mitosis and was a new critical player in PLK1 kinetochore localization. NCAPG2 localization and PLK1 recruitment to the kinetochores of misaligned chromosomes are critical for the completion of proper chromosome alignment. Polo‐like kinase 1 is a well‐established mitotic regulator with a diverse range of biologic functions continually being identified throughout the cell cycle 15. In addition, PLK1 is highly up‐regulated and is a predictor for poor prognosis in a large number of human cancer types 16, 17, 18, 19, 20. Notably, overexpression of PLK1 has been shown to stimulate cell proliferation and oncogenic transformation 21, while PLK1 depletion/inhibition triggers apoptosis in various cancer cell lines 22, 23, and impairs tumour growth in mice 24. However, the expression profile and functional role of NCAPG2 in human cancer remains largely unknown.

In this study, we found that NCAPG2 expression was significantly increased in NSCLC tissues compared to adjacent normal lung tissues, and was closely associated with tumour progression and poor prognosis in lung AD patients. In addition, knockdown of NCAPG2 significantly inhibited the proliferation of lung AD cells *in vitro via* inducing G2/M phase arrest. Based on these results, we proposed that increased NCAPG2 expression might be important in lung AD carcinogenesis and inhibition of NCAPG2 could be a promising therapeutic target in lung AD.

## Materials and methods

### Patients and tissue samples

Paired adjacent normal lung tissue and NSCLC were obtained from 21 patients who underwent primary surgical resection of NSCLC in Jinling Hospital, Nanjing University School of Medicine between March 2013 and November 2013. Following surgical removal, the tissue samples were immediately stored in liquid nitrogen prior to total RNA extraction. All tissues were histopathologically confirmed as NSCLC or adjacent normal lung tissue. No patient had received pre‐operative adjuvant therapy and all patients signed an informed consent form under protocols approved by the Institutional Review Board of Jinling Hospital, Nanjing University School of Medicine.

### Immunohistochemical staining and scoring in tissue microarrays

Commercial tissue microarrays (HLug‐Ade180Sur‐01 and HLug‐Ade030PG‐01; Shanghai Outdo Biotech, Shanghai, China) of 105 lung AD patients and matched adjacent normal lung tissue were used to evaluate expression of NCAPG2. Antigen retrieval was performed with high pressure for 5 min., using citrate buffer, pH 6.0. The sections were incubated overnight with the primary antibodies at 4°C (Rabbit polyclonal Anti‐NCAPG2 antibody, HPA026631; Sigma‐Aldrich, St. Louis, MO, USA). Negative controls were performed by replacing the primary antibody with PBS. Finally, the slides were analysed separately by two pathologists without knowing the patients’ clinical information. The staining intensity was scored on a scale of 0–3 as negative (0), weak (1), medium (2) or strong (3). The extent of the staining, defined as the percentage of positive staining areas of tumour cells in relation to the whole tumour area, was scored on a scale of 0 (0%), 1 (1–25%), 2 (26–50%), 3 (51–75%) and 4 (76–100%). An overall protein expression score (overall score range, 0–12) was calculated by multiplying the intensity and positivity scores.

### Cell culture

Three lung AD cell lines (A549, SPC‐A1 and NCI‐H1299), one lung squamous carcinomas cell line (NCI‐H1703) and a normal human bronchial epithelial cell line (HBE) were purchased from the Institute of Biochemistry and Cell Biology of the Chinese Academy of Sciences (Shanghai, China). A549, NCI‐H1299 and NCI‐H1703 cells were cultured in RPMI 1640 medium, and SPC‐A1and HBE cells were cultured in DMEM (GIBCO, Invitrogen, Carlsbad, CA, USA) medium supplemented with 10% foetal bovine serum (FBS), 100 U/ml penicillin, and 100 mg/ml streptomycin (Invitrogen) at 37°C and 5% CO_2_.

### RNA extraction and qPCR assays

Total RNA was extracted from the cultured cells or tissues using TRIZOL reagent (no.15596‐026; Invitrogen), according to the manufacturer's instructions. Total RNA (1 μg) was reverse transcribed in a final volume of 20 μl using random primers under standard conditions for the PrimeScript RT reagent Kit (RR036A; TaKaRa, Dalian, China). Quantitative reverse‐transcription polymerase chain reaction (qRT‐PCR) was done using SYBR Premix Ex Taq II (Perfect Real Time) (RR420A; TaKaRa) according to the manufacturer's instructions. The specific primers used are presented in Table 1. The PCR reaction was conducted at 95°C for 30 sec. followed by 40 cycles of 95°C for 5 sec. and 60°C for 35 sec. in the ABI 7500 real‐time PCR system (Applied Biosystems, Foster City, CA, USA). Results are presented as mean ± S.D. for duplicate runs. The relative quantification of NCAPG2 expression was calculated using the 2^−∆∆CT^ method relative to β‐Actin.

**Table 1:** The primers used in qPCR analysis

Primer name	Primer sequence 5′–3′
NCAPG2‐F	AACCAAGCCAACATCTCCAG
NCAPG2‐R	AAATCCCACCCTTTCCCTATT
siNCAPG2‐1	CAGCCUAAAUGAAUUACUATT UAGUAAUUCAUUUAGGCUGTT
si‐NCAPG2‐2	GCGUAUCCAUCAAGCUUUATT UAAAGCUUGAUGGAUACGCTT
si‐NCAPG2‐3	GCCAAACUUUACACGAUUATT UAAUCGUGUAAAGUUUGGCTT
Actin‐F	TGACGTGGACATCCGCAAAG
Actin‐R	CTGGAAGGTGGACAGCGAGG

### Western blotting

Cells were lysed using a lysis buffer containing the mammalian protein extraction reagent RIPA (Beyotime, Nantong, China), a protease inhibitor cocktail (Roche, Basel, Switzerland) and PMSF (Roche). The protein concentration was detected using a Bio‐Rad protein assay kit. Samples containing 40 μg of protein from two different cell lines were electrophoresed on a 10% SDS‐PAGE and then transferred onto 0.22‐μm nitrocellulose membranes (Sigma‐Aldrich) and incubated with specific antibodies. The ECL chromogenic substrate was used to detect specific bands. Protein expression was quantified using densitometry (Quantity One Software; Bio‐Rad, Hercules, CA, USA), with β‐Actin used as a control. Information on the antibodies is provided: NCAPG2 (1:500 dilution, Sigma‐HPA026631, lot no. A81365; Sigma‐Aldrich); P27 (1:1000 dilution, CST‐3686 (Cell Signaling Technology, Danvers, MA, USA), lot no. P46527); P21(1:1000 dilution, sc‐397 (Santa Cruz BIotechnology, TX, USA), lot no. P38936); Cdc2 (1:1000 dilution, CST‐9116, lot no. P06493); CylcinB1 (1:1000 dilution, CST‐4138, lot no. P14635); β‐actin (1:1000 dilution, CST‐4970, lot no. P60709).

### RNA interference by siRNA

A549 and SPC‐A1 cells were plated and cultured in growth media until cell density reached 50–60% prior to small interfering RNAs (siRNAs) transfection. When transfecting cells with siRNA, Lipofectamine 2000 (Invitrogen, Shanghai, China) were employed according to the manufacturer's instructions. The sequences of the siRNAs were presented in Table 1. At 48 hrs post transfection, cells were harvested for qPCR or western blot analysis.

### Cell proliferation assays and colony formation assay

Control siRNA‐ or NCAPG2 siRNA‐transfected A549 and H1299 cells (3000/well) were allowed to grow in 96‐well plates 24 hrs after siRNA transfection. Cell proliferation was monitored every 24 hrs using the Cell Proliferation Reagent Kit I (MTT; Roche Applied Science). All of the experiments were performed in quadruplicate. For the cells to form colonies, a total of 750 transfected control siRNA‐ or NCAPG2 siRNA‐transfected A549 and H1299 cells were placed onto a fresh six‐well plate and maintained in media containing 10% FBS, replacing the medium every 4 days. After 2 weeks, the colonies were fixed with methanol and stained with 0.1% crystal violet (Sigma‐Aldrich). The visible colonies were manually counted. Triplicate wells were assessed for each treatment group.

### Flow‐cytometric analysis of apoptosis and the cell cycle

Following the double staining with FITC‐Annexin V and Propidium Iodide (PI), the cells were analysed by flow cytometry (FACScan^®^; BD Biosciences, San Jose, CA, USA) equipped with CellQuest software (BD Biosciences). Cells were categorized as early apoptotic cells, late apoptotic cells, dead cells or viable cells. The ratio of early apoptotic cells to late apoptotic cells was compared to that for controls from each experiment. For the cell cycle analysis, the cells were stained with PI using the CycleTESTTM PLUS DNA reagent kit (BD Biosciences) according to the manufacturer's protocol. The ratio of cells in the G0/G1, S and G2/M phases were counted and compared. All of the samples were assayed in triplicate.

## Results

### Up‐regulation of NCAPG2 expression in NSCLC tissues

**Figure 1:**
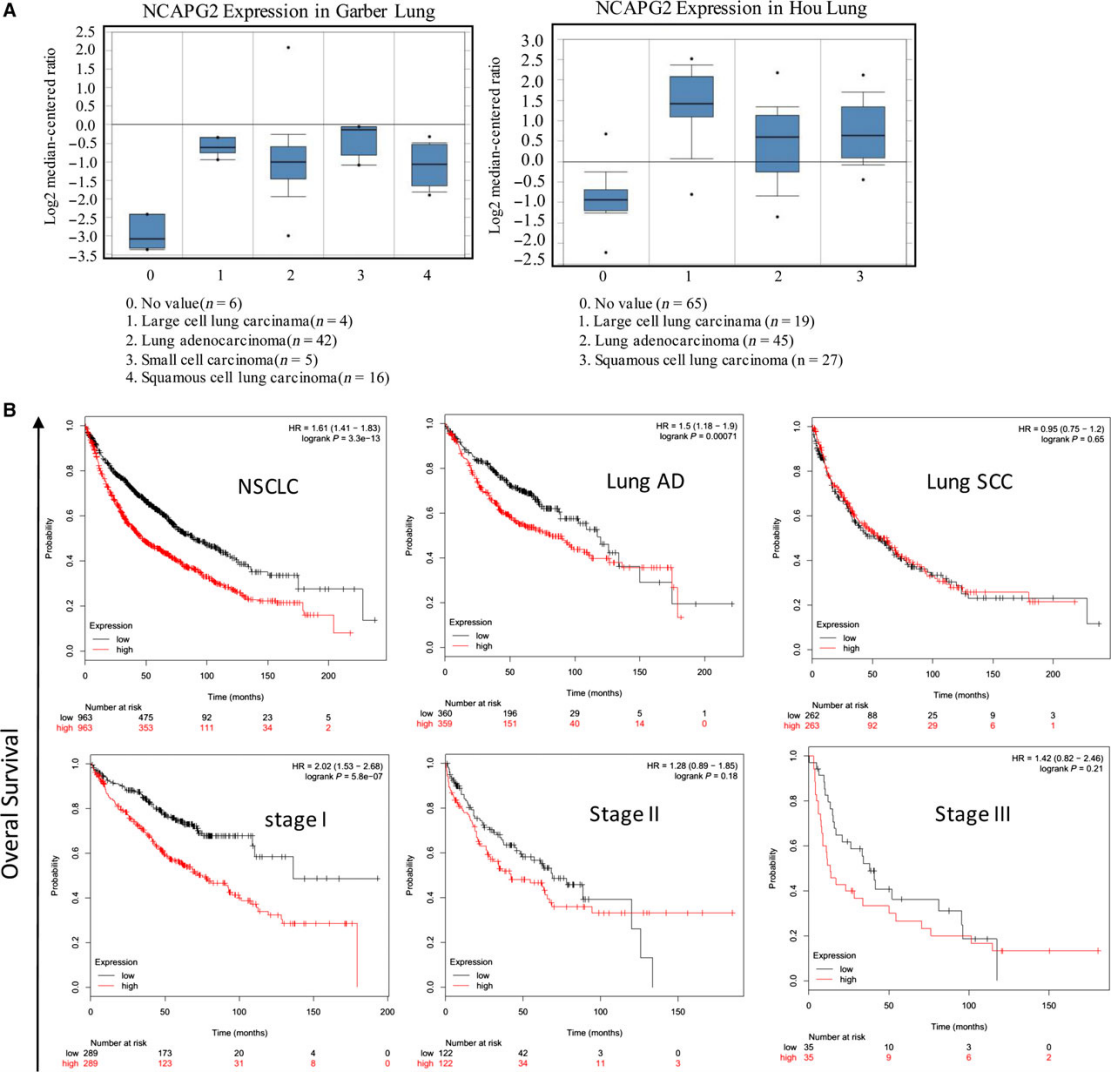
NCAPG2 is highly up‐regulated in human NSCLC. (**A**) NCAPG2 gene expression is highly up‐regulated in NSCLC compared with that in normal lung tissues. Microarray data analyses of NCAPG2 gene expression in human lung cancer tissues and normal lung (Hou 25, and Garber 26 are plotted. The Student's *t*‐test was conducted using the Oncomine software (www.oncomine.org). The boxes represent the 25th through 75th percentiles. The horizontal lines represent the medians. The whiskers represent the 10th and 90th percentiles, and the asterisks represent the end of the ranges. (AD: adenocarcinoma; LCC: large cancer lung carcinoma; SCC: squamous carcinoma). (**B**) NCAPG2 high expression is associated with poor survival in NSCLC. Kaplan–Meier plots of overall survival: comparison of patients with high *versus* low expression of NCAPG2 in NSCLC patients stratified by different histological types and disease stages. The Kaplan–Meier plots were generated by Kaplan–Meier Plotter (http://www.kmplot.com). Patients with high NCAPG2 expression had a worse OS and among NSCLC, lung AD and stage I, but not in lung SCC, stage II or stage III.

We first systematically examined NCAPG2 mRNA expression between NSCLC and normal pulmonary samples in the publicly available database Oncomine. We carried out an expression analysis of NCAPG2 using two microarray data sets from Hou and Garber lung cancer cohorts downloaded from Oncomine 25, 26. In both cohorts, the expression of NCAPG2 mRNA was significantly increased in NSCLC tissues compared to normal tissues (Fig. 1A). Nest, we evaluated the prognostic effect of NCAPG2 mRNA expression by Kaplan–Meier Plotter analysis (www.kmplot.com) in the publicly available database. Kaplan–Meier plots of overall survival (OS; Fig. 1B) indicated that NCAPG2 mRNA high expression is associated with poor survival in NSCLC. When stratified by different histological types and disease stages, patients with high NCAPG2 mRNA expression had a worse OS among lung AD and stage I, but not in lung squamous carcinoma, stage II or stage III.

**Figure 2:**
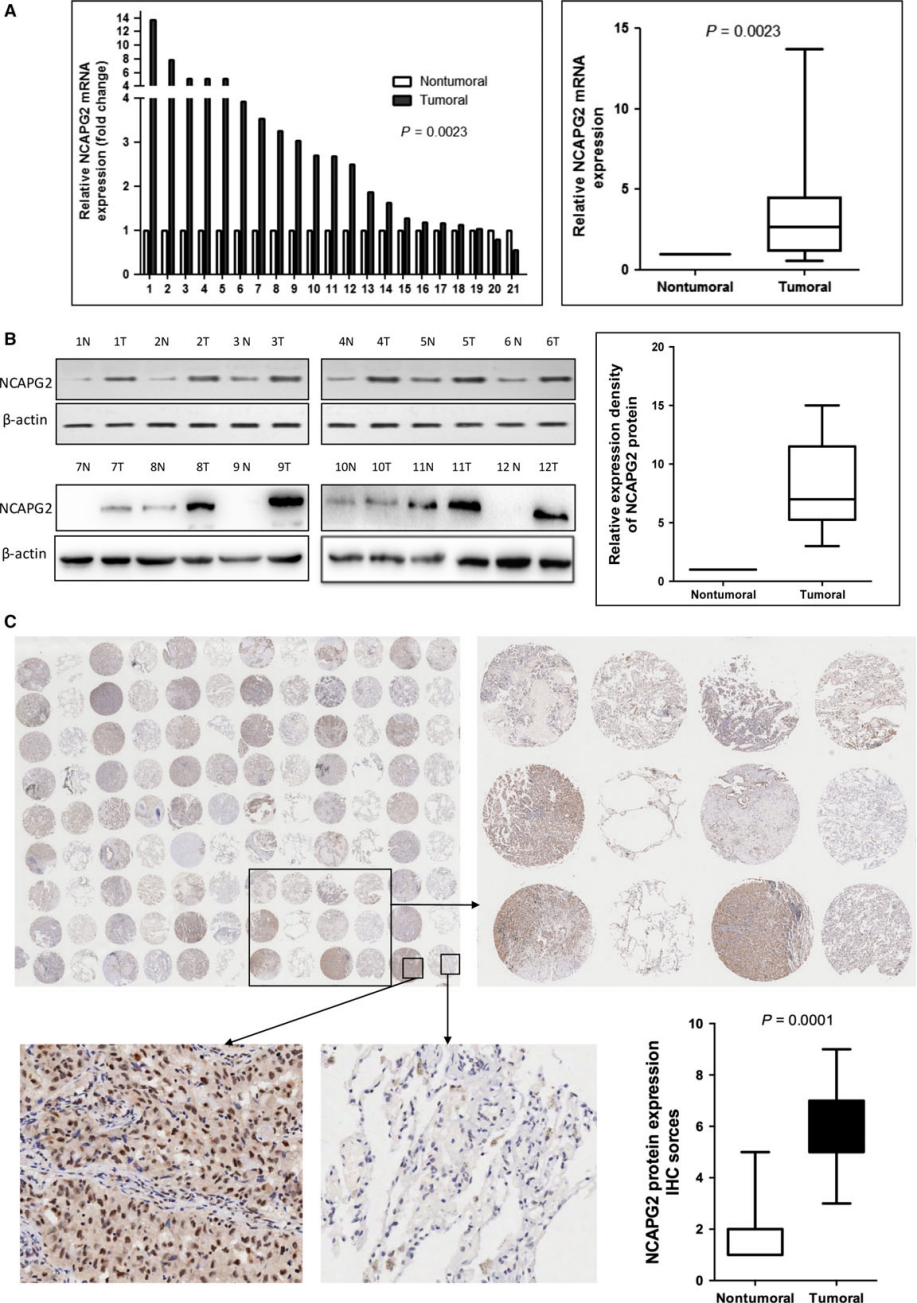
NCAPG2 is up‐regulated in primary lung cancer tissues. (**A**) Relative expression levels of NCAPG2 mRNA in NSCLC tissues and paired adjacent non‐tumoral tissues (*n* = 21) by qRT‐PCR. The levels of NCAPG2 in NSCLC tissues are significantly higher than those in non‐tumoral tissues. (**B**) Expression levels of NCAPG2 protein in 12 paired primary NSCLC tissues were determined by Western blotting. Quantitative analysis of relative expression density is shown in right panel. Each column represents the mean ± S.D. (N: Non‐tumoral; T: tumour). (**C**) Expression levels of NCAPG2 protein in 105 paired lung AD tissues and paired adjacent non‐tumoral tissues were determined by immunohistochemistry (IHC). Expression of NCAPG2 protein in lung cancer tissues and non‐tumoral lung tissues is shown with different magnification. Statistical analysis of NCAPG2 scores is shown in lower panel(right).

Furthermore, a total of 21 paired clinical NSCLC tissues and adjacent normal tissues were analysed for NCAPG2 mRNA expression using qRT‐PCR, NCAPG2 expression was significantly up‐regulated in cancerous tissues (*P* = 0.0023; Fig. 2A). In addition, Western blot analysis showed an increased level of NCAPG2 protein in nearly all of the 12 paired NSCLC tissues compared the adjacent normal tissues.

We then determined NCAPG2 protein expression in clinical samples using immunohistochemistry (IHC) analysis in 105 pairs of lung AD carcinoma and matched adjacent normal tissues. The immunostaining NCAPG2 protein was predominantly located in nuclear. According to the staining location, the NCAPG2 protein expression score (overall score range, 0–12) was calculated by multiplying the intensity and positivity scores. As shown in Figure 2C, the expression level of NCAPG2 protein in lung AD tissues were significantly higher than those in corresponding non‐tumour tissues (*P* = 0.0001). These data demonstrated that the up‐regulation of NCAPG2 might play critical roles in lung AD development and progression.

### Correlation between NCAPG2 overexpression and clinicopathological factors and survival of lung AD patients

**Figure 3:**
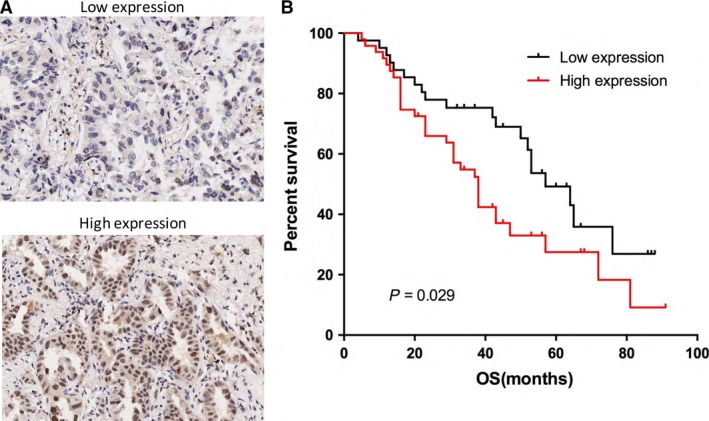
Overexpression of NCAPG2 protein associated with poor overall survival in patients with lung AD. (**A**) IHC staining of low expression and high expression of NCAPG2 in 105 patients with lung AD were shown. (**B**) Kaplan–Meier curves for overall survival rate in 90 patients with lung AD according to the expression status of NCAPG2. The median staining score (6.2) was served as a cut‐off to divide patients into high and low NCAPG2. Black line: patients with low NCAPG2expression, red line: patients with high NCAPG2 expression. High NCAPG2 protein expression was associated with significantly decreased overall survival (*P* = 0.029, log‐rank test).

To determine the significance of NCAPG2 overexpression in lung AD patients, 90 patients were divided into two groups according to the mean expression level (staining scores). The correlations of NCAPG2 overexpression and clinicopathological factors of lung AD patients are assessed and displayed in Table 2. For NCAPG2 expression, ≤6.2 was defined as low expression, >6.2 was high expression (Fig. 3A). The difference by statistical analyses indicated that a high expression of NCAPG2 protein was also found to be significantly correlated with lymph node metastasis (*P* = 0.038) and pathologic‐Tumour Nodes Metastasen stage (p‐TNM stage) (*P* = 0.024). There was no significant association between NCAPG2 expression and other clinicopathological features.

**Table 2:** Correlation of NCAPG2 protein expression with various clinicopathological features in 90 patients with lung adenocarcinoma

	Number of patients	NCAPG2 protein expression		
		Low (≤6.2)	High (>6.2)	*P*‐value†
All patients	90	48	42	
Gender				
Male	49	25	24	0.308
Female	41	23	18	
Age (years)				
<65	51	27	24	0.295
≥65	39	21	18	
Size of tumour				
≤3 cm	32	16	16	0.201
>3 cm	58	32	26	
Lymph node metastasis (pN)				
N0	39	28	11	0.038*
N1–3	51	20	31	
p‐TNM stages				
I	30	20	10	0.024*
II+III	60	28	32	

^*^ *P* < 0.05. ^†^Chi‐square test.

We further evaluated the prognostic significance of NCAPG2 overexpression in lung AD patients. From Kaplan–Meier survival curves, we observed that patients with high expression of NCAPG2 protein survived a shorter survival time than patients with low levels (*P* = 0.029, log‐rank test, Fig. 3B). For further investigation, we analysed the prognostic factors by a multivariate Cox regression model (Table 3). The results indicated that N stage (HR = 1.591, 95% CI = 1.205–2.829, *P* = 0.036), p‐TNM Stage (HR = 1.573, 95% CI = 1.261–2.671, *P* = 0.034) and high expression of NCAPG2 protein (HR = 2.107, 95% CI = 1.361–3.37, *P* = 0.028) were observed to an independent prognostic factor for AD patients.

**Table 3:** Multivariate analyses of NCAPG2 protein expression and other clinical prognostic factors in lung adenocarcinoma patients

Factors	Over survival
	HR (95% CI), *P*
Age(≥65/<65 years)	0.752 (0.466–1.571), 0.235
Gender (Femle/Male)	1.461 (0.845–2.526), 0.453
Size of tumour (>3 cm/≤3 cm)	1.321 (0.745–1.972), 0.202
N stage (N1‐3/N0)	1.591 (1.205–2.829), 0.036^a^
p‐TNM Stage (II+III *versus* I)	1.573 (1.261–2.671), 0.034^a^
NCAPG2 expression (high/low)	2.107 (1.361–3.37), 0.028^a^

a *P* < 0.05. HR: hazard ration; 95% CI: 95% confidence interval.

### The effect of NCAPG2 on cell proliferation on lung adenocarcinoma *in vitro*

**Figure 4:**
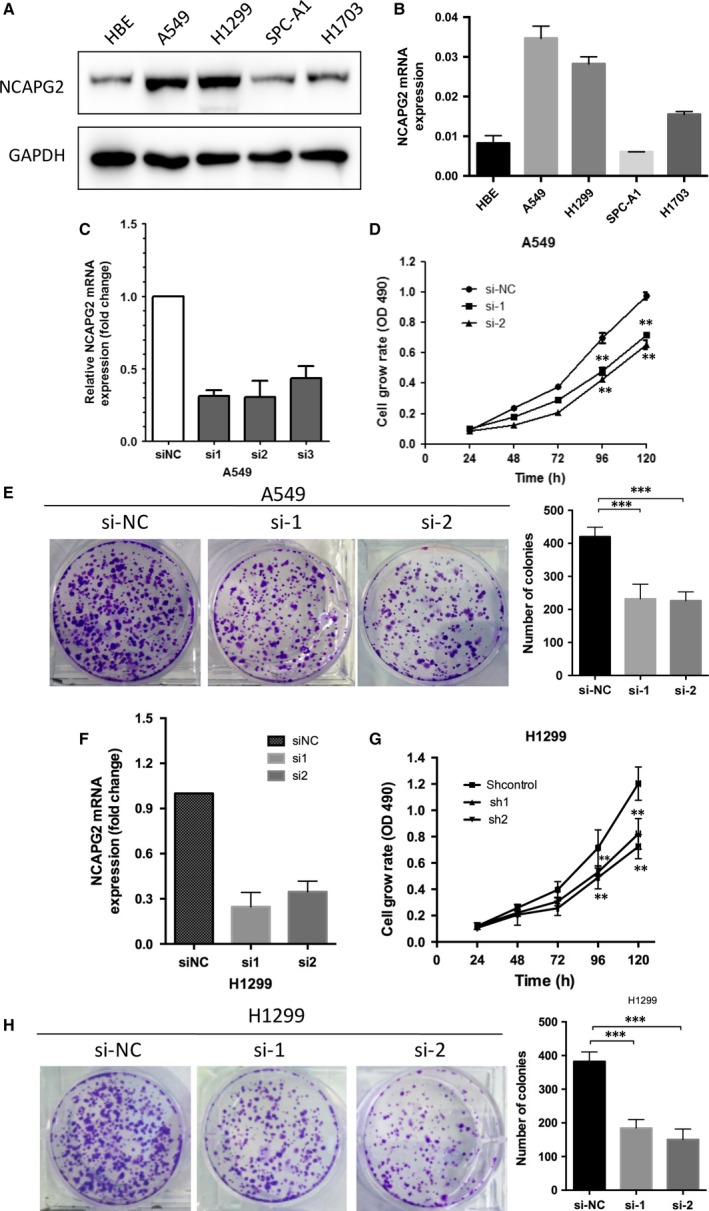
Effect of NCAPG2 gene silencing on the growth of lung adenocarcinoma cells. (**A** and **B**) Expressions of NCAPG2 were detected in NSCLC cell lines (HBE, A549, H1299, SPC‐A1and H1703) by Western blot analysis (**A**) and qRT‐PCR (**B**). β‐Actin was used as a loading control. (**C**) qRT‐PCR analysis of NCAPG2 expression level in A549 transfected with three discrete chemically synthesized siRNAs or scramble(si‐NC). (**D**) MTT assay was performed to determine the proliferation of A549 transfected with scramble (si‐NC) or siRNA NCAPG2 (si‐1 or si‐2). (**E**) A colony formation assay of A549 cells transduced with scramble (si‐NC) or siRNA NCAPG2 (si‐1 or si‐2). The colonies were counted and captured. The data represent the mean ± S.D. from three independent experiments. **P < 0.01, ***P < 0.005. (**F**) qRT‐PCR analysis of NCAPG2 expression level in H1299 transfected with three discrete chemically synthesized siRNAs and scramble (si‐NC). (**G**) MTT assay was performed to determine the proliferation of H1299 transfected with si‐NC and siRNA NCAPG2 (si‐1 or si‐2). (**H**) A colony formation assay of H1299 cells transduced with si‐NC and siRNA NCAPG2 (si‐1 or si‐2). The colonies were counted and captured. The data represent the mean ± S.D. from three independent experiments. **P < 0.01, ***P < 0.005.

We performed qRT‐PCR and Western blot analysis to determine the expression level of NCAPG2 in six human NSCLC cell lines which include both squamous carcinoma and AD. It was determined that NCAPG2 expression was elevated in two lung AD cell lines (A549 and H1299) and lung squamous carcinoma (H1703), whereas NCAPG2 expression was lower in HBE cells and AD cell lines (SPC‐A1) (Fig. 4A and B). To investigate the mechanism by which NCAPG2 contributed to malignancy of lung AD, we used chemically synthesized siRNAs to knock down endogenous NCAPG2 in A549, H1299 and H1703. At 48 hrs post transfection, NCAPG2 expression levels were knocked down more than 55–70% in these three cells relative to negative control transfected cells. The results showed NCAPG2‐siRNA1,2 transfected cells exhibited significantly reduced NCAPG2 transcripts compared with cells transfected with scramble (si‐NC) (Fig. 4C and F, Fig. S1A).

Then, we examined the impact of NCAPG2 knockdown on the proliferation of human lung AD cells. Compared to the control siRNA transfection, NCAPG2 siRNA led to a significant decrease in these three cells’ viability as monitored by an MTT assay (Fig. 4D and G, Fig. S1B). Additionally, NCAPG2 knockdown also substantially reduced the colony‐forming ability in these three cells (Fig. 4E and H, Fig. S1C). These results suggested that NCAPG2 might be critical for lung AD cell proliferation.

### siRNA‐mediated knockdown of NCAPG2 leads to G2/M cell cycle arrest

**Figure 5:**
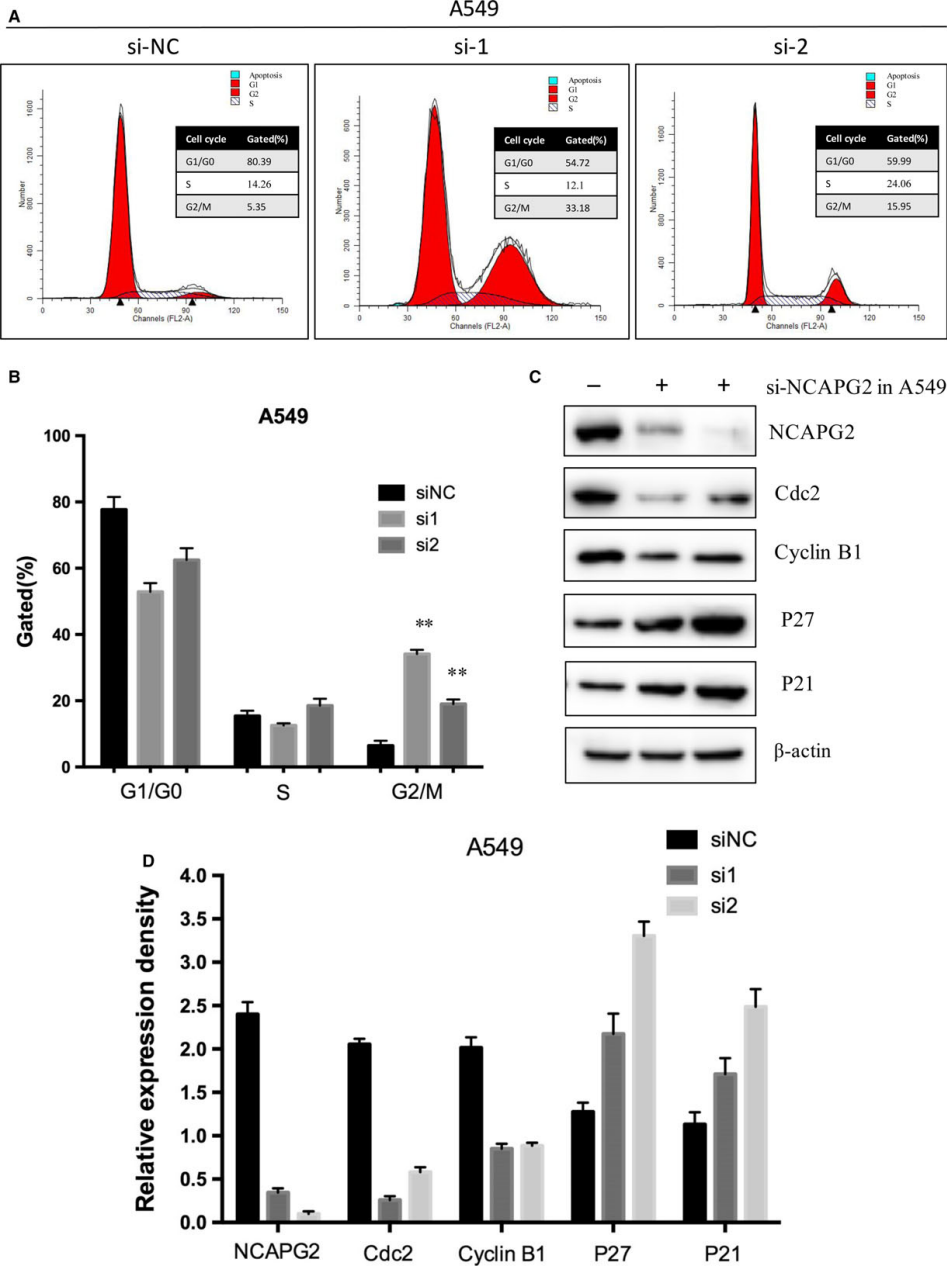
The effects of NCAPG2 on NSCLC cell cycle *in vitro*. (**A** and **B**) A549 cell was transfected with si‐NC and siRNA NCAPG2 (si‐1 or si‐2). The DNA content was quantified by flow cytometric analysis. Representative fluorescence activated cell sorting images and statistics based were presented. The data represent the mean ± S.D. from three independent experiments. (**C** and **D**) Western blot analysis of NCAPG2 and G2/M transition‐related proteins in A549 cell was transfected with si‐NC and siRNA NCAPG2 (si‐1 or si‐2). β‐actin protein expression was used as an internal control. The data represent the mean ± S.D. from three independent experiments. **P < 0.01.

To further examine the role of NCAPG2 knockdown in cell cycle, we investigated the cell cycle distribution of siRNA‐NCAPG2 or control siRNA transfected A549 and H1703 using flow cytometry. In NCAPG2‐siRNA transfected cells A549, the proportion of cells in G2 phase increased by 27.4% in cells treated with si‐1 and 12.4% in cells treated with si‐2 (Fig. 5A and B). In addition, flow cytometry indicated that G2/M phase arrest was also found in NCAPG2‐siRNA transfected cells H1703 (Fig. S1D). Furthermore, to investigate the molecular mechanism underlying the influence of NCAPG2 on cell cycle in lung AD cell, we focused on key associated regulators of G2/M phase transition including Cyclin B1, Cdc2, p21, and P27 27, 28. Concordantly, knockdown of NCAPG2 decreased the expression of Cyclin B1 and Cdc2, which are known to regulate G2/M phase transition. In contrast, the protein expression of cyclin‐dependent kinase inhibitor (CKI) p27 and P21 were markedly increased (Fig. 5C and D). Altogether, our results demonstrated that NCAPG2 contributes greatly to the development of NSCLC cell proliferation and leads to G2/M phase arrest, possibly through alterations of p27, p21 and G2/M phase cell cycle‐related protein expression (Cyclin B1, Cdc2).

### NCAPG2 expression positively correlated with PLK1 expression in human lung adenocarcinoma tissues

**Figure 6:**
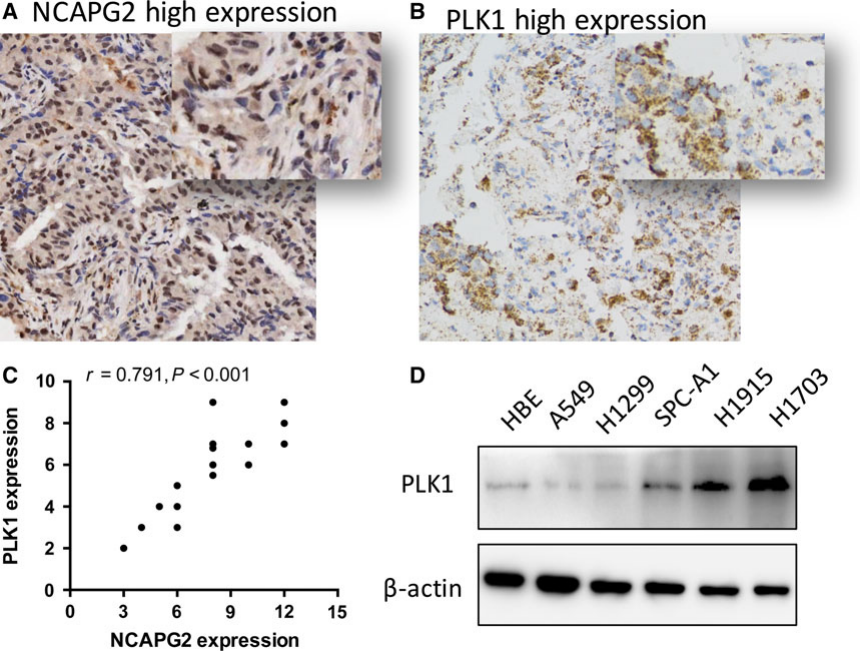
NCAPG2 expression positively correlated with PLK1 expression in human lung adenocarcinoma tissues. (**A** and **B**) NCAPG2 and PLK1 expression level in 20 lung adenocarcinoma tissues were detected by IHC. (**C**) The expression levels of NCAPG2 positively correlated with PLK1 level. (**D**) The expression level of PLK1 in NSCLC cell lines and HBE cell lines was examined by Western blot analysis.

The study by Kim *et al*. 14 showed that NCAPG2 could contribute to chromosome segregation by interacting with PLK1 during mitosis. To confirm the relationship between NCAPG2 and PLK1 expression level in lung AD, we determined the expression level of NCAPG2 and PLK1 in 20 lung AD tissues (Fig. 6A and B). Pearson's correlation analysis found that the expression levels of NCAPG2 positively correlated with PLK1 level in human lung AD tissues (*r* ^2^ = 0.791, *P* < 0.001, Fig. 6C). In addition, the expression level of PLK1 in NSCLC cell lines and HBE cell lines was conducted by Western blot analysis, which indicated that PLK1 expression was elevated in NSCLC cell lines and lower in HBE.

## Discussion

The chromosomal condensin complex is a major molecular effector of chromosome condensation and segregation in diverse organisms ranging from bacteria to humans 4. NCAPG2 is a subunit of condensin II complex, which is regarded as a key player in mitotic chromosome condensation 5. Previous study found that NCAPG2 plays an important role in regulating proper chromosome segregation through a functional interaction with PLK1^14^. Importantly, PLK1 could act as an oncogene by promoting proliferation in human cancer. Although the function of NCAPG2 during mitosis has been well defined, the expression status, functional role and underlying mechanism of NCAPG2 during oncogenesis and cancer proliferation remain unclear.

Our studies demonstrated for the first time the expression profile and functional role of NCAPG2 in human cancer. Our data provided information that NCAPG2 mRNA and protein expression were significantly increased in NSCLC tissues compared to the paired normal lung tissues. The data from online available gene expression‐profiling data sets of lung cancer was consistent with our finding. We found that high expression of NCAPG2 protein was correlated with lymph node metastasis and p‐TNM stages and contributed to adverse clinical outcome in lung AD patients. The prognostic effect of NCAPG2 expression was further confirmed by Kaplan–Meier Plotter analysis in the publicly available database. Furthermore, knockdown of NCAPG2 significantly inhibited lung AD cell proliferation and G2/M phase arrest *in vitro*. Taken together, the observations of this study indicated that NCAPG2 may serve as an oncogene and may play an important role in lung AD development and progression.

This study demonstrated that depletion of NCAPG2 resulted in G2 phase arrest through alterations in the expressions of p27, p21 and G2/M phase transition‐related proteins (Cyclin B1, Cdc2), suggesting that NCAPG2 plays an important role during cell cycle in lung AD cells. The activation and inactivation of Cdc2/Cyclin B complex were the hallmark of cell cycle at the G2/M phase 29. On the other hand, p21and p27, which belong to CKIs, interact with cyclin‐dependent kinases, and act as an inhibitor of cell cycle progression 30, 31. In the present study, transfection with si‐NCAPG2 in lung AD cell caused the accumulation of the CKIs p21 and p27, and reduction in Cdc2 and Cyclin B expression, leading to G2/M phase arrest. It is plausible that alterations in cell cycle‐associated proteins may lead to the arrest of G2/M phase in lung AD cell cells with knockdown of NCAPG2.

In summary, our investigations found that NCAPG2 was overexpressed in NSCLC and may act as a prognostic factor in lung AD. Moreover, inhibition of NCAPG2 suppressed cancer cell proliferation *via* leading to G2/M phase arrest through alterations of p27, p21, Cyclin B1 and Cdc2 expression. Based on the cancer‐specific expression and the functional characteristics, our results indicated that NCAPG2 could serve as a potential prognosis marker and a novel therapeutic target for NSCLC patients.

## Conflicts of interest

The authors confirm that there are no conflicts of interest.

## Supporting information


**Figure S1** Effect of NCAPG2 gene silencing on the growth of H1703. (**A**) qRT‐PCR analysis of NCAPG2 expression level in H1703 transfected with three discrete chemically synthesized siRNAs or scramble (si‐NC). (**B**) MTT assay was performed to determine the proliferation of H1703 transfected with scramble (si‐NC) or siRNA NCAPG2 (si‐1 or si‐2). (**C**) A colony formation assay of H1703 cells transduced with scramble (si‐NC) or siRNA NCAPG2 (si‐1 or si‐2). The colonies were counted and captured. The data represent the mean ± S.D. from three independent experiments. *P < 0.05, **P < 0.01, ***P < 0.005. (**D**) H1703 cell was transfected with si‐NC and siRNA NCAPG2 (si‐1 or si‐2). The DNA content was quantified by flow cytometric analysis. The data represent the mean ± S.D. from three independent experiments.
