# Supplementary figures and images for: NCAPG2 promotes tumour proliferation by regulating G2/M phase and associates with poor prognosis in lung adenocarcinoma
Source: J Cell Mol Med. 2016 Nov 15;21(4):665–76. doi: 10.1111/jcmm.13010 (PMC5345611; doi:10.1111/jcmm.13010)

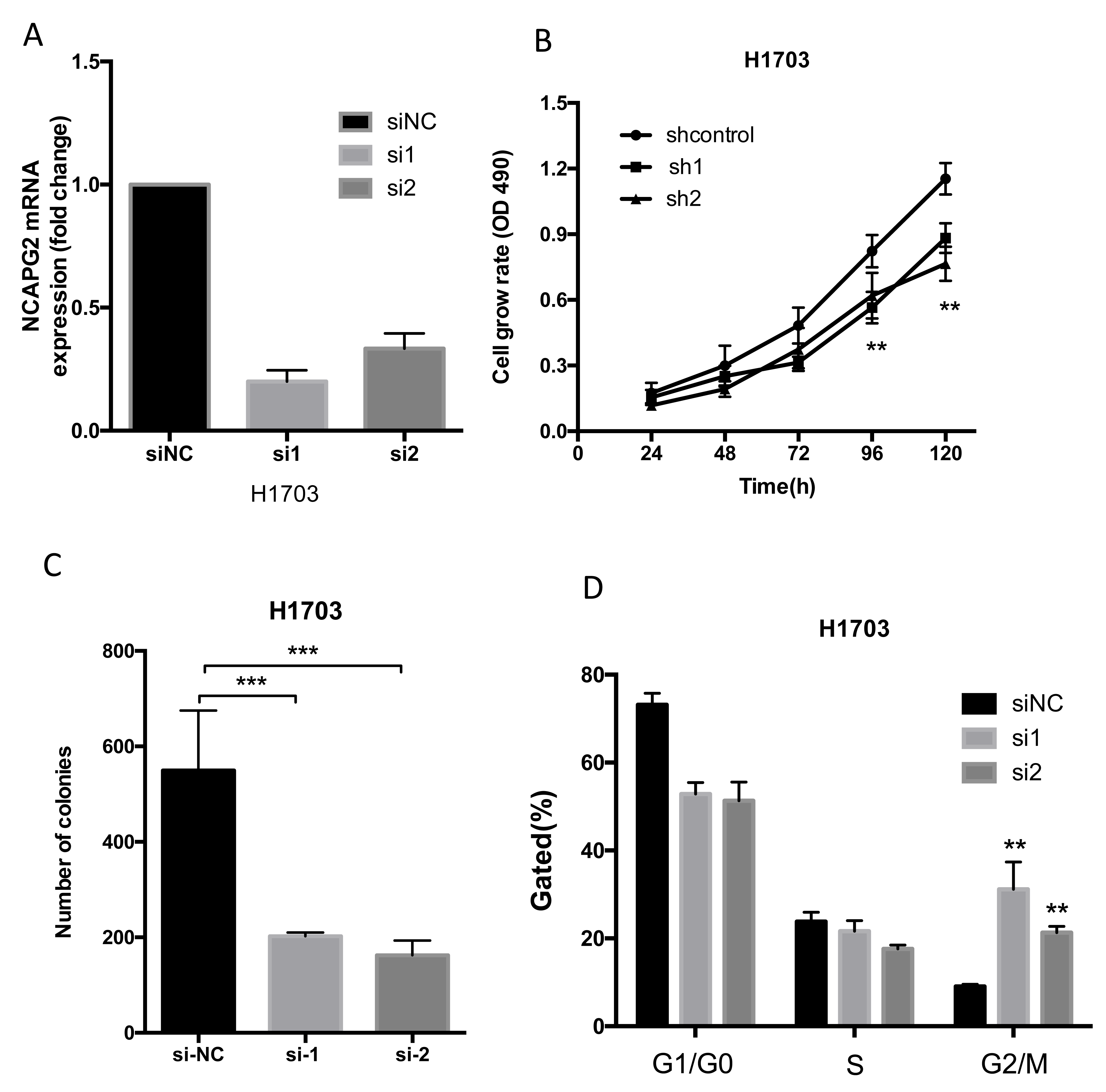

Supplement: Supplementary file 1 — Figure S1 Effect of NCAPG2 gene silencing on the growth of H1703. (A) qRT‐PCR analysis of NCAPG2 expression level in H1703 transfected with three discrete chemically synthesized siRNAs or scramble (si‐NC). (B) MTT assay was performed to determine the proliferation of H1703 transfected with scramble (si‐NC) or siRNA NCAPG2 (si‐1 or si‐2). (C) A colony formation assay of H1703 cells transduced with scramble (si‐NC) or siRNA NCAPG2 (si‐1 or si‐2). The colonies were counted and captured. The data represent the mean ± S.D. from three independent experiments. *P < 0.05, **P < 0.01, ***P < 0.005. (D) H1703 cell was transfected with si‐NC and siRNA NCAPG2 (si‐1 or si‐2). The DNA content was quantified by flow cytometric analysis. The data represent the mean ± S.D. from three independent experiments. [file JCMM-21-665-s001.tif]
